# Knowledge, attitudes and beliefs regarding colorectal cancer screening among ethnic minority groups in the Netherlands – a qualitative study

**DOI:** 10.1111/hex.12428

**Published:** 2015-11-17

**Authors:** Anke J. Woudstra, Evelien Dekker, Marie‐Louise Essink‐Bot, Jeanine Suurmond

**Affiliations:** ^1^ Department of Public HealthAcademic Medical CentreUniversity of AmsterdamAmsterdamThe Netherlands; ^2^ Department of Gastroenterology and HepatologyAcademic Medical CentreUniversity of AmsterdamAmsterdamThe Netherlands

**Keywords:** attitudes, beliefs, colorectal cancer, colorectal cancer screening, ethnic minorities, prevention, health belief model, knowledge, qualitative research

## Abstract

**Background:**

Research has shown that ethnic minority groups are less likely to participate in colorectal cancer (CRC) screening than the majority population and hence less likely to be diagnosed at an early stage when treatment is potentially more successful.

**Objective:**

To explore knowledge, attitudes and beliefs regarding CRC and CRC screening among ethnic minority groups in the Netherlands.

**Design:**

We conducted qualitative interviews with 30 first‐generation immigrants born in Turkey, Morocco and Surinam. We based the topic guide on the health belief model. Framework analysis was used to analyse our data.

**Results:**

Although knowledge of CRC and CRC screening was limited, all respondents felt susceptible to CRC. CRC screening was perceived to mainly benefit those individuals with poor health and symptoms. Although most respondents had a positive attitude towards CRC screening, knowledge about its potential harms was limited and self‐efficacy to participate was low. Adult children acted as important mediators in providing access to information. The language barrier and low literacy formed serious barriers to informed participation in CRC screening.

**Conclusion:**

To ensure that all eligible individuals, including ethnic minority groups, have equal opportunities to informed participation in screening, targeted communication strategies should be developed, such as oral and visual channels, and face‐to‐face communication in the mother tongue. This will help ethnic minority groups to make an informed decision about participation in CRC screening.

## Background

In Europe, colorectal cancer (CRC) is the second most common cause of cancer with over 200 000 deaths per year. Worldwide, CRC ranks third in new cases of cancer and fourth in cancer deaths, with an estimated 1.3 million cases and more than 600 000 annual deaths. Population‐based CRC screening has proven to be effective in reducing CRC incidence and mortality.^1^

### Dutch CRC screening programme

In the Netherlands, a national population‐based CRC screening programme has been implemented since 2014. Biennial screening by faecal immunochemical test (FIT) is offered free of charge to all individuals from 55 to 75 years of age. This CRC screening programme is introduced in phases, with expected nationwide coverage in 2019. Invitees are instructed to perform the test at home by dipping the sampling stick into four different parts of the stool sample. FIT‐based screening programmes have shown a higher sensitivity for CRC and its precursor lesions (polyps) than the guaiac‐based faecal occult blood test (gFOBt). Additionally, FIT‐based CRC screening programmes showed a higher participation rate than gFOBT‐based CRC screening.^2^

If the FIT result provides evidence for blood in the stool sample above a certain cut‐off value, participants are offered further diagnostic work‐up through colonoscopy. All eligible individuals are sent an invitation package by postal mail that includes the FIT and information materials in the Dutch language only, but with a reference in English, Turkish and Arabic to the website of the Dutch National Institute for Public Health and the Environment (RIVM) for information in other languages.

The initial numbers from the first year of the Dutch CRC screening programme showed 71.3% (529 056 people) screening uptake across the general Dutch population.^3^ Figures about uptake gradients across socio‐economic and ethnic groups have not been published yet. However, one Dutch study by Deutekom *et al*.,^4^ analysing the uptake rate in the implementation pilot of the Dutch CRC screening programme by ethnicity, showed that participation among ethnic minority groups was significantly lower than among ethnic Dutch. Previous research from other Dutch cancer screening programmes also showed lower screening uptake rates among ethnic minority groups than among the general Dutch population.^5^

Over the coming decades, the total number of ethnic minority elderly will rapidly increase in the Netherlands. In this study, we use the standard definition of Statistics Netherlands for the classification of ethnic groups: a person is considered to be of non‐Dutch ethnic origin if s(he) was born abroad and at least one of the parents was born abroad (first generation). The utility of ‘country of birth’ in this definition has become widely accepted for identifying ethnic groups in the Netherlands.^6^ Among the largest groups of ethnic minority elderly in the Netherlands are those from Turkish, Moroccan and Surinamese origin (see Table 1 for a description of these ethnic minority groups). These three groups account each for about 2% of the total population, including about 396000 (2.4%) inhabitants from Turkey, about 375000 (2.2%) inhabitants from Morocco and about 348000 (2.1%) inhabitants from Surinam (Statistics Netherlands, 2014).^7^ A large proportion of ethnic minority elderly have lower educational level, low mastery of Dutch and low health literacy which may contribute to difficulty with obtaining, communicating, processing and understanding basic health information and making informed health decisions.^8^

**Table 1:** Background on ethnic minority elderly from Turkish, Moroccan and Surinamese origin in the Netherlands

Turkish, Moroccan and Surinamese immigrants are among the largest ethnic minority groups in the Netherlands. About half of all Moroccans and Surinamese and one‐third of all Turkish immigrants live in one of the four main cities (Amsterdam, The Hague, Rotterdam or Utrecht) in the Netherlands.
Turkish and Moroccan men started coming to the Netherlands in the late 1960s and 1970s as guest workers. What was meant to be a temporary stay, turned into a permanent stay from the 1970s to the 1990s, as partners and children came to the Netherlands for reunification. Most Moroccan and Turkish elderly people have low socio‐economic status, low educational levels and low proficiency in Dutch. The majority is Muslim.
Surinam is a former Dutch colony (‘Dutch Guyana’) in South America. With the independence of Surinam in 1975, a large group of Surinamese moved to the Netherlands. The official language in Surinam is Dutch. Most Surinamese are therefore proficient in Dutch.

### Previous research

Previous research in other countries showed that ethnic minority groups are less likely to participate in CRC screening programmes than the majority population and hence less likely to be diagnosed at an early stage when treatment is potentially more successful.^9^, ^10^, ^11^, ^12^ Several UK studies reported language barriers and cultural barriers to CRC screening among ethnic minority groups in the UK (e.g. Indian, Pakistan Bangladeshi, Caribbean, African and Chinese).^10^, ^12^ However, most of the studies on barriers to CRC screening among ethnic minority groups have been conducted in the USA.^13^ Findings of American studies cannot easily be generalized to the Netherlands or other European settings because of differences in funding of health care, the organization of population‐based CRC screening and the diversity in ethnic minority groups. For example, lower participation in CRC screening is often associated with lower health insurance coverage in the USA and CRC screening in the USA is often visit based, requiring a patient to visit a doctor and a doctor to offer CRC screening.^9^

### Study aim

It remains unclear which factors influence CRC screening uptake among ethnic minority groups in the Netherlands. Therefore, our aim was to understand current knowledge, attitudes and beliefs regarding CRC and CRC screening among the three largest ethnic minority groups in the Netherlands (i.e. Turks, Moroccans and Surinamese, see table 1) in the context of the start of the Dutch population‐based CRC screening programme.

## Methods

### Ethics approval

According to Dutch law, this study was waived from requiring ethical approval. We ensured that we were open about the purpose of our research with respondents, that the anonymity of the respondents was guaranteed by the use of codes and that we obtained written informed consent in advance from all respondents.

### Selection and recruitment of respondents

We used a purposive sampling strategy to recruit the respondents during February–July 2014. Respondents aged 48–75 were eligible for participation because they spanned the ages of being eligible for CRC screening (ages 55–75). Only two Turkish female respondents were under the age of 55 (ages 48 and 53) and were included as a result of snowball sampling.^14^

The Turkish (*n = *10) and Moroccan respondents (*n = *11) were recruited through the network of a Dutch organization for the health of immigrants (‘Stichting Gezondheid Allochtonen Nederland’ [SGAN]). SGAN is an organization that revolves around the work of volunteers and offers advice, support and health promotion to vulnerable ethnic minority elderly with the aim to improve their lives.

Two bilingual female Turkish interviewers and one bilingual female Moroccan interviewer with experience in qualitative research, who were all part of SGAN, were added to the research team. They were instructed about the aims of the research and their role and received an interview training. Recruitment was carried out via telephone and face‐to‐face by these three interviewers.

Surinamese respondents (*n *=* *9) were recruited through their general practitioner, using the network of general practitioners at the Academic Medical Centre (AMC) in Amsterdam. The respondents were invited by a letter at their home address. For participation, they could fill in an rsvp card with name, telephone number and email address. The first author (AW) contacted them for an interview.

Due to the recent start (January 2014) and the long‐term phased introduction of the screening programme, none of the respondents in this study had received an invitation for CRC screening yet. All respondents received a 10 Euro gift voucher for their participation in our study. Interviews were conducted over a 6‐month period (February–July) in 2014 and were held at respondents’ private homes or inside the AMC in Amsterdam, according to the preference of the respondent. We stopped recruitment when no new themes were identified in the data and when theoretical saturation was achieved.^15^

### Data collection

The topic guide, based on the health belief model (HBM), included the following six domains: perceived susceptibility, perceived severity, perceived benefits, perceived barriers, self‐efficacy and cues to action (see table 2 for explanation of the HBM constructs and the topic guide).^16^ For example, to examine the perceived susceptibility, we asked the following question: ‘Who do you think is more likely to develop CRC?’. The topic guide was discussed and adapted with the Moroccan and Turkish interviewers. AW conducted the interviews with Surinamese respondents in Dutch. The Turkish and Moroccan interviewers conducted the interviews in the mother tongue of the Turkish and Moroccan respondents. At the beginning of each interview, respondents were given the invitation package and the interviewers provided them with a brief explanation of CRC screening. Respondents shared their responses to the information materials and how they might use the information materials if they were to receive an invitation. After the interviews, the interviewers shared their interpretation of what respondents said and reflected on the interviews with AW. All nine interviews with Surinamese respondents and eleven interviews with Moroccan and Turkish respondents were audiotaped and transcribed verbatim. In 10 cases, the interviews were not audiotaped because respondents did not consent to the taping of the interviews and extensive notes were made. The interviews lasted about 45–90 min. The interviews conducted in a foreign language were translated to Dutch by the Turkish and Moroccan interviewers.

**Table 2:** Topic guide based on the health belief model

HBM constructs	Explanation of constructs	Primary questions in topic guide
Perceived susceptibility^a^	Beliefs about the risks of getting a condition	What do you think are the causes of CRC?
		Who do you think is susceptible for CRC?
		Who do you think is more likely to develop CRC?
		Are you more or less likely to develop CRC, compared to other people?
		When it comes to lowering one's risks of getting CRC, what do you think can be done?
Perceived severity^a^	Beliefs about the seriousness of a condition and its consequences	When I say cancer, what thoughts come to mind?
		When I say CRC, what thoughts come to mind?
		Do you know someone in your community with cancer? How would you react to this person?
Perceived benefits	Beliefs about the effectiveness of taking action to reduce risk or seriousness	Have you heard of CRC screening? Can you tell me what you think CRC screening is?
		What is the most important reason for you to participate or not participate in CRC screening?
		How do you think that people in your community would feel about CRC screening?
Perceived barriers	Beliefs about psychological costs of taking action	What do you think is the most unpleasant about CRC screening?
Cues to action	Factors that activate ‘readiness to change’	How do you think people are best reached with information materials about CRC and CRC screening?
		What would you make more likely to understand the information materials about CRC and CRC screening?
		Would you participate in CRC screening to you, if your GP would inform you about CRC screening?
		With whom would you discuss the information materials about CRC and CRC screening?
		Do you think other people find it important for you to participate?

a Perceived susceptibility and perceived severity are often labelled together as perceived threat.

### Data analysis

We analysed our data using a framework approach. The framework approach is a deductive matrix‐based approach to qualitative data analysis in which *a priori* data are structured according to key themes and subthemes within an overall framework.^17^ The aim of the framework approach is to summarize and classify data within a thematic framework.^14^ This approach allowed us to incorporate the constructs of the HBM within our analysis. However, to ensure that we were not forcing our data within the predetermined constructs of the HBM, we adopted a two‐stage approach to our analysis.^18^ The benefit of this approach is to sensitize researchers to relevant issues, processes and interpretations that might not necessarily be identified using an inductive approach.^18^

In the first stage, we used inductive thematic analysis. The first transcripts were independently manually coded and agreed on by two researchers (AW and JS). This was an iterative process; memos of the key issues and themes were saved, and interviews were reread to identify themes in the data. In regular meetings with AW, JS, ML and the Turkish and Moroccan interviewers, codings and interpretation of the data were discussed. In the second stage, we used qualitative data analysis software (MAXQDA, Version 11, VERBI GmbH, Berlin, Germany) to map the emergent themes onto constructs of the HBM. We reread the interviews and applied codes to the whole data set by devising a chart, which helped us to summarize the data. Finally, using the constant comparative method, we examined similarities and differences between the data and our coding within and between the transcripts.^19^

## Results

Characteristics of respondents are summarized in Table 3. We present our findings using the constructs of the HBM.^16^ *Perceived threat* illustrates participants’ beliefs about their own chances of getting CRC (i.e. perceived susceptibility) and their beliefs about the seriousness of CRC (i.e. perceived severity). The theme *perceived benefits* illustrates participants’ beliefs about the effectiveness of participating in CRC screening to reduce their risk of CRC. The theme *perceived barriers* illustrates participants’ beliefs about the costs of participating in CRC screening. *Self‐efficacy* illustrates participants’ confidence in their ability to perform the FIT and to undergo CRC screening, and finally, the last theme presents *cues to action*, that is lay recommendations to increase accessibility of CRC screening information among ethnic minority groups.

**Table 3:** Characteristics of respondents

Respondents	Country of origin	Sex	Age	Length of stay in the Netherlands (years)	Education
R01	Surinam	F	65	39	Secondary school
R02	Surinam	F	65	39	Secondary school
R03	Surinam	M	63	39	Secondary school
R04	Surinam	M	64	34	Secondary school
R05	Surinam	M	62	34	Secondary school
R06	Surinam	F	67	46	Secondary school
R07	Surinam	M	68	40	Secondary school
R08	Surinam	F	59	34	Secondary school
R09	Surinam	M	60	43	Not documented
R10	Morocco	F	74	38	None
R11	Morocco	F	60	Missing	None
R12	Morocco	M	60	44	Primary school
R13	Morocco	F	55	38	Primary school
R14	Morocco	F	63	42	None
R15	Morocco	F	64	35	None
R16	Morocco	M	59	34	Secondary school
R17	Morocco	M	67	Missing	Primary school
R18	Morocco	M	63	45	Primary school
R19	Morocco	F	59	36	Primary school
R20	Morocco	M	63	38	Secondary school
R21	Turkey	F	64	26	None
R22	Turkey	F	60	40	None
R23	Turkey	F	58	30	None
R24	Turkey	M	55	36	Higher general secondary education
R25	Turkey	M	67	40	Primary school
R26	Turkey	F	61	42	Primary school
R27	Turkey	F	53	41	Primary school
R28	Turkey	F	55	28	Primary school
R29	Turkey	F	63	14	Primary school
R30	Turkey	F	48	30	Primary school

### Perceived threat of CRC

All respondents reported to feel susceptible to CRC and perceived this disease as very serious. Due to its association with death, CRC was often perceived to be more serious than other chronic diseases that were believed to be part of everyday life. Respondents in all three groups repeatedly stated that cancer caused a lot of fear. The discussion of CRC was therefore widely avoided:

We talk about health but not about serious diseases. That is a taboo within our community. You just don't talk about it. But we do talk about diabetes, high blood pressure, cholesterol. We avoid serious diseases. (Moroccan woman, R11)

‘Non‐natural’ food (i.e. processed food) was perceived as the most important cause of CRC across all three ethnic groups. Accordingly, many respondents had an overwhelming sense of ‘what can you do?’ when discussing their perceptions on susceptibility of CRC.

Nowadays, there is nothing that you can do about CRC because everything has become non‐natural. Nowadays, there is no way to find natural food. (Turkish man, R25)

Respondents’ answers to questions on their personal risk for CRC were often ‘I don't know’ or ‘Everyone is at risk for CRC’. Although beliefs about the causes of cancer differed between respondents, the majority of the answers carried a sense of powerlessness or being in the hands of ‘fate’ or ‘luck’.

Nobody knows what causes CRC but I did hear and read that everyone is born with malign and benign cells but CRC develops in one person but it won't in another person. You just need to have bad luck. (Surinamese man, R07)

However, the perception of fate did not disempower respondents to participate in CRC screening. On the contrary, many respondents, irrespective of ethnicity, felt encouraged to participate in CRC screening in accordance with their religious beliefs:

Our faith says: do everything you can, and then leave it up to Allah. Therefore, you need to participate in CRC screening. Then you have done everything you could do and if you are then diagnosed with CRC, it is given by Allah, and you have to accept it. (Turkish man, R25)

This was also often described to be the common belief in their communities. Yet, there was one respondent who found participation in CRC screening meaningless, expressing fatalistic views on CRC:

I don't see the need to participate, and besides, if Allah wants me to have CRC, then I should have it. (Moroccan woman, R14)

### Perceived benefits of participation in CRC screening

Most respondents expressed positive attitudes towards CRC screening. The most commonly stated benefit was early detection of CRC:

Imagine that you will get something like that [CRC], then it will be discovered early. I find that very important and it's very good that that is being done. (Surinamese woman, R02)

Many respondents asked questions about why CRC screening could not be introduced before the age of 55 years and why participation in CRC screening is not obligatory. However, many respondents with a positive attitude only recognized the benefits of CRC screening and dismissed its potential harms, such as the risk of a false‐negative FIT result:

That is why they do the examination, right? If they can't find anything, well then I won't have it. (Turkish woman, R28)

Some respondents believed that their participation in CRC screening would benefit others. Their accounts illustrated misconceptions about CRC screening being a scientific study that could improve the health of others in the future:

I would participate in the CRC screening programme anyway. With other scientific studies in which I participated, there were even no benefits for myself but more for the future. People could be helped with these studies. I also participated in those scientific studies. Thus, that was not for my own benefit. For me, it would mean nothing, but it will for people in the future. (Surinamese man, R03)

### Perceived barriers of participation in CRC screening

Prior awareness of CRC screening was low, especially among Turkish and Moroccan respondents. When the interviewers showed the invitation package, the majority expressed unawareness. Respondents suggested that this unawareness might lead to mistrust towards CRC screening:

Because otherwise you might think that this is another activity to earn money or we are used as guinea pigs because you hear that a lot; that if you don't have symptoms, research is being done to test medicines. If you don't know anything about it then you will be afraid for it or you don't want to participate. (Turkish woman, R29)

Lack of symptoms was mentioned as a key barrier to participation in CRC screening among Turkish and Moroccan respondents. This perceived barrier illustrated a misconception about the nature of CRC screening, which was often understood to diagnose cancer, rather than looking for a polyp or early stage CRC:

Because I do not have symptoms, I think that those screenings are useless. As long as I feel healthy and don't have any symptoms, I will not participate. (Turkish woman, R28)

On the other hand, having symptoms or poor health was an important motivator to participate in CRC screening for many respondents:

Cancer is a terrible disease and I want to know if I have something in my intestines. I am often constipated, so I want to know if I have something. (Turkish woman, R21)

Only after CRC screening was explained by the interviewers, some respondents expressed that they were more aware, not only of the positive aspects but also of possible negative aspects of CRC screening and that it may generate even more ill health:

If you need to do a colonoscopy that already has risks, why would I take that risk? Why make a healthy intestine sick? No thank you, I already have enough diseases. (Turkish woman, R27)

Finally, while collecting stool samples for the FIT was not considered to be a major barrier, the discussion of faeces produced a lot of embarrassment:

Talking about faeces is just like talking about sex. It is difficult for children to talk about this with their parents. (Moroccan woman, R11)

One respondent mentioned that being found out to have collected stool samples for the FIT could be unsettling:

I don't want to participate because you need to go through all this effort to put all that in a bottle and then you need to mail it. What if I forget to put it in the mailbox? Then it will start to smell. And what if someone opens the fridge and finds a bottle with faeces? (Turkish woman, R27)

### Self‐efficacy

Many Turkish and Moroccan respondents stated that they were afraid to ‘fail’ the FIT and that they would not be able to complete the test on their own. Consequently, in order for them to participate, they would need their children to be their eyes, ears and hands:

I really need my children's help or else I am afraid that I will do something wrong and it would be a shame of the material. I will wait until she is able to come, then I will discuss it with her and we will do it together and then she will also mail it for me. (Turkish man, R25)

Children also played an important role in the final decision about participation in screening:

I find their [the children's] opinions very important. If they tell me not to participate, I will not participate. (Moroccan woman, R10)

### Lay recommendations for increasing accessibility to CRC screening information

Respondents suggested informing elderly migrants about CRC screening in places where they regularly meet, such as community centres, schools, mosques and Dutch assimilation courses:

As long as there is sufficient promotion [Moroccan people will participate]. So for Moroccan people, they will go to the Mosque and [Moroccan] women, they will go to schools [to pick up their children]. (Moroccan woman, R11)

In addition, many respondents stated that if they were advised by their general practitioner (GP) to participate in CRC screening, they would absolutely participate. In fact, having no GP recommendation gave respondents the impression that participation was not as important.

I also participated in breast cancer screening because I received an invitation and my GP recommended it. (Turkish woman, R26)

More involvement of the GP in the screening procedure might also increase respondents’ self‐efficacy:

So, he [my GP] can tell me what to do next. (Moroccan woman, R19)

Respondents voiced a need for visual and verbal information materials in their mother tongue rather than written information, as many Turkish and Moroccan respondents, in particular women, were not able to write or read in their mother tongue. In addition, respondents emphasized the value of word of mouth advertising in their communities to create public awareness:

In our community, people follow each other very much. So if one is positive towards CRC screening, the rest will also be positive. (Moroccan woman, R15)

I think that if a person comes to explain the information, then people [Surinamese community] would participate. Yes, if people hear from other people that they participated then they will also participate. (Surinamese man, R07)

Many Turkish and Moroccan respondents were not effectively reached by the Dutch media campaign on the Dutch CRC screening programme that had been going on preceding our data collection. None used the Internet to access health information. However, respondents did often seek and access health information in their mother tongue, mentioning television and radio. For example, one Turkish woman (R29) explained that she discovered early stage breast cancer because she had seen information about breast cancer on a Turkish medical television channel.

Although cancer was not easily discussed within the three communities, respondents in all three groups reported that talking about CRC within their communities would contribute to the normalization of screening. With this, one Moroccan woman (R11) referred to the Dutch population‐based breast cancer screening programme:

With that breast cancer screening programme, there was so much publicity, it became normal to go. (Moroccan woman, R11)

Cancer is most certainly remains taboo. If we receive information, I think it will become less of a taboo. (Surinamese women, R06)

## Discussion

This is the first study to explore knowledge, attitudes and beliefs regarding CRC and CRC screening among the three largest ethnic minority groups in the Netherlands of Turkish, Moroccan and Surinamese origin in context of the start of the Dutch CRC screening programme. Respondents had limited knowledge about CRC and CRC screening. Despite this limited knowledge of CRC, perceived seriousness of CRC was high. Contrary to previous research that showed that ethnic minority groups considered themselves to be at low risk for CRC,^11^ all respondents felt susceptible for CRC. This high perceived susceptibility to CRC might be linked to their understanding of risk factors of CRC. Respondents commonly mentioned fate and wider availability of ‘non‐natural’ food as most important risk factors. Notably, these fatalistic attitudes towards CRC did not reflect barriers to participation. While respondents believed that CRC is completely beyond an individual's control, the majority expressed willingness to participate in CRC screening, in accordance with their religious beliefs. Our findings suggest that fatalistic attitudes may be a facilitator to CRC screening participation. This finding contrasts the explanation of lower participation rates in CRC screening due to fatalistic attitudes.^20^ These conflicting findings may be explained by different interpretations of fate among ethnic minority groups.

One of the major perceived barriers was lack of symptoms. Conversely, respondents with poor health or symptoms showed higher interest in CRC screening. This supports previous research that showed that people with symptoms are more likely to have positive attitudes towards CRC screening.^21^ Respondents thus perceived CRC screening as an activity that would mostly benefit individuals with poor health or symptoms. This finding suggests a lack of understanding about the preventive nature of CRC screening and the need for emphasizing the necessity of CRC screening in the absence of symptoms.

We identified low mastery of Dutch, which is common among first‐generation migrants in the Netherlands, as the most important barrier to CRC screening. The written Dutch information materials and the online available translated information materials did not adequately convey the information needed to make an informed decision about participation. As the objectives of CRC screening remained unclear, respondents expressed mistrust of the purpose of CRC screening. Mistrust has earlier been identified as a major barrier to participation in CRC screening.^22^

Screening invitees need to have an adequate understanding of both the potential benefits and harms to be able to make an informed decision about participation. We found that respondents in all ethnic groups held misconceptions about CRC screening and were inadequately informed about its potential harms and benefits. Even though the majority of the respondents had positive attitudes towards participation, few were aware of the risk of false‐negative FIT results. This might eventually lead to false reassurance and being less aware of symptoms.^23^

Respondents in all three groups voiced a desire for verbal and visual information to better understand the potential harms and benefits of CRC screening. Communication strategies targeted to their language characteristics and limited knowledge of CRC and CRC screening is needed to ensure equal opportunities to participation in CRC screening. Previous research also found that people from countries with another mother tongue than the majority population preferred verbal or visual channels, or face‐to‐face communication.^21^

Turkish and Moroccan respondents had low self‐efficacy and were highly dependent on family members for participation in CRC screening. Children in particular acted as important mediators in providing access to information. We were encouraged to find that talking about CRC and CRC screening in the mother tongue increased awareness and self‐efficacy. However, we also found that this reliance on others presented further barriers as children might have their own agendas, not see the relevance of CRC screening or be reluctant to talk about faeces and cancer with their parents.

### Practice implications

Easier and accessible information, such as verbal and visual information in the mother tongue, might help to understand both the potential benefits and harms of CRC screening and enable ethnic minority groups to make an informed decision about participation. While at present, general practitioners (GPs) are not involved in the screening invitation strategy, wider involvement of GPs might increase the self‐efficacy of ethnic minority groups and enable addressing sensitive topics, rather than needing to ask help from others. Hence, respondents might be more likely to participate in CRC screening when they have the opportunity to discuss the decision whether or not to participate with a health care provider. As family members often play an important role in decision making within these groups, attention should be given to the difficulties that arise from the dependence on others to take part in CRC screening. Information materials also need to be targeted at adult migrant children, who can pass on relevant information to their parents.

### Limitations

Several limitations of our study should be considered. First, the HBM is more descriptive than explanatory and does not necessarily suggest strategies for changing preventive health behaviours, such as screening. However, the recommendations to increase CRC screening are a necessary first step to develop interventions for optimizing the accessibility of CRC screening information materials for specific groups, including ethnic minority groups. Second, although we did find some overlap between the themes, similar to other research,^12^, ^24^ we found that the HBM is a workable model for exploring the factors that are associated with CRC screening participation among ethnic minority groups because it offers a theoretical framework to understand barriers to CRC screening in an integrated and inclusive way.^12^ Third, by using an organization for the health of migrants (SGAN) and a network of general practitioners in Amsterdam for the recruitment of respondents, it is possible that the study population was biased towards people with a positive attitude towards CRC screening. In addition, we did not explore factors influencing CRC screening participation among ethnic Dutch individuals. Some of our findings may also be applicable for Dutch individuals and may not be specific for ethnic minorities. Some caution is therefore needed in generalizing our findings. Lastly, we decided to use trained bilingual interviewers and it is known that control may be lost over these interviews. For example, despite having a topic guide, the Moroccan and Turkish interviewers may not have prompted on those issues that seemed most obvious to them. Further, recruitment of Turkish male respondents had limited success due to the fact that the Turkish interviewers were female and because of a reluctance to discuss sensitive topics such as ‘cancer’ and ‘faeces’ between men and women. Still, by using bilingual interviewers, we managed to interview a large group of ethnic minority elderly; a group generally hard to reach in research due to language barriers, illiteracy and mistrust of research.^25^ In addition, we found that conducting interviews in the language of choice of the respondents created an environment in which they were generally able to freely ask questions and discuss sensitive topics such as cancer and faeces. We realize that ethnic minorities are not a homogenous group. Yet, few ethnic‐specific differences were found within the constructs of the HBM in this study. Our finding that the three ethnic minority groups had limited knowledge of CRC and CRC screening is confirmed by a Dutch FIT‐based CRC screening pilot assessing knowledge and attitudes towards CRC screening in the Netherlands.^26^ In this Dutch study, people of non‐Dutch ethnicity (i.e. Turkish, Moroccan, Afro‐Caribbean and other) had significantly less knowledge than the general Dutch population. However, in our study, low awareness of CRC screening and limited knowledge might also partly be explained by the recent start of the CRC screening programme. Yet, it seems plausible that a language barrier is one of the main causes of impeded access to information about CRC screening for many ethnic minority elderly, not just in the Netherlands as comparable barriers have been described for example in the UK.^10^, ^12^ We therefore believe that our findings can be applied in other countries to improve the access to cancer screening information for ethnic minority elderly, especially those with low mastery of the language of the host country and lower educational level.

## Conclusion

To ensure that all eligible individuals, including ethnic minority groups have equal opportunities to informed participation in CRC screening, information materials should be targeted to language characteristics and use of channels of communication. Specifically, limited knowledge of CRC and CRC screening, low self‐efficacy and reliance on others to complete the FIT should be taken into consideration when developing effective and accessible communication strategies.

## Author contributions

A.J. Woudstra, E. Dekker, J. Suurmond and M.L. Essink‐Bot contributed to the design and the revision of the article. A.J. Woudstra, N. Lale (SGAN), K. Azzouz (SGAN) and G. Avsar (SGAN) contributed to the data collection. A.J. Woudstra and J. Suurmond contributed to the analysis. A.J. Woudstra, J. Suurmond, M.L. Essink‐Bot, N. Lale (SGAN), K. Azzouz (SGAN) and G. Avsar (SGAN) contributed to the interpretation. A.J. Woudstra is responsible for the draft of the article. All authors have read and approved the article.

## Conflict of interest

The authors declare no conflict of interest.
